# Hospital costs of different treatment strategies for anastomotic leakage after total mesorectal excision: a multicentre cost analysis

**DOI:** 10.1007/s10151-025-03215-2

**Published:** 2025-10-08

**Authors:** D. J. Nijssen, K. Wienholts, M. J. Postma, W. A. Bemelman, J. Tuynman, W. Laméris, P. J. Tanis, R. Hompes

**Affiliations:** 1 Department of Surgery, Amsterdam UMC Location University of Amsterdam, Amsterdam, the Netherlands; 2 Cancer Center Amsterdam, Treatment and Quality of Life, Amsterdam, The Netherlands; 3 Cancer Center Amsterdam, Imaging and Biomarkers, Amsterdam, The Netherlands; 4 Department of Health Sciences, University of Groningen, University Medical Center Groningen, Groningen, The Netherlands; 5 Department of Economics, Econometrics and Finance, Faculty of Economics and Business, University of Groningen, Groningen, The Netherlands; 6 Department of Pharmacology and Therapy, Universitas Airlangga, Surabaya, Indonesia; 7 Center of Excellence in Higher Education for Pharmaceutical Care Innovation, Universitas Padjadjaran, Bandung, Indonesia; 8 Department of Surgical Oncology and Gastrointestinal Surgery, Erasmus MC, Rotterdam, the Netherlands

**Keywords:** Anastomotic leakage, Total mesorectal excision, Rectal cancer, Hospital costs, Treatment strategies, Cost analysis

## Abstract

**Background:**

Limited data exist on hospital costs incurred by anastomotic leakage (AL), particularly in relation to specific treatment approaches. This study aimed to analyse the incremental hospital costs of AL after total mesorectal excision (TME), stratified by treatment strategy, over a 1-year time horizon.

**Methods:**

Patients undergoing total mesorectal excision (TME) for rectal cancer (2020–2023), included in the control cohort of the IMARI-study at 15 Dutch centres, were analysed. A cost analysis was conducted according to Dutch National Healthcare Institute guidelines. The primary outcome was the incremental hospital costs incurred by patients with AL detected within 30 days postoperatively, stratified by treatment strategy.

**Results:**

The analysis compared treatment costs in 32 patients with AL and 82 patients without AL. The average hospital costs per patient in the first postoperative year were €15.312. In patients with AL, the mean incremental costs were €24.333. Major cost drivers in the AL group were prolonged hospitalization (+€13.150) and (re)interventions (+€8.910). The treatment costs differed significantly between strategies: no faecal diversion (€10.062), faecal diversion with passive drainage (€23.903), faecal diversion with active drainage (€35.552), and salvage surgery (€38.793).

**Conclusions:**

AL after TME resulted in a nearly fourfold increase in hospital costs compared with patients without AL. Salvage surgery was the most expensive treatment strategy, followed by faecal diversion with active drainage. Future studies should evaluate how these treatment costs relate to clinical success rates, including rates of chronic pelvic sepsis and permanent stomas.

**Trial registration:**

This study used data from the IMARI-study. The IMARI-study is registered with the Dutch Central Committee on Research Involving Human Subjects (NL67600.018.18) and is submitted to the http://www.onderzoekmetmensen.nl/en database (NL-OMON26456 and NL-OMON55903).

**Supplementary information:**

The online version contains supplementary material available at 10.1007/s10151-025-03215-2.

## Introduction

Despite advances in surgical techniques, anastomotic leakage (AL) after restorative rectal cancer resection still occurs in up to 25% of patients [1]. AL has a significant impact on postoperative morbidity, long-term oncologic outcomes, definitive stoma rates, and even mortality [1–5]. Despite extensive literature on risk factors and interventions related to AL, the incidence rates have shown little reduction over the years [6]. Anastomotic leakage causes prolonged hospitalization, intensive care unit (ICU) admissions, readmissions, and eventually high-risk salvage procedures, all of which impose a major financial burden on both hospitals and society.

The hospital costs of AL after restorative colorectal resections reported in the current literature range between €2.250 and €83.633, varying significantly depending on the healthcare setting and country [7]. Societal costs, such as those incurred by patients and their families and those in other sectors outside healthcare, including costs caused by productivity losses, have not been reported but are likely to form an equally significant burden. In addition, a decrease in health-related quality of life resulting from AL can severely impact patients and may also influence societal costs [8]. The wide range of costs reported in different studies is partly attributable to the heterogeneity of methodologies used in health economic evaluations [7, 9]. Initiatives such as the Consolidated Health Economic Evaluation Reporting Standards (CHEERS) and the Consensus Health Economic Criteria (CHEC) aim to enhance transparency and standardization in reporting for health economic evaluations, but they are not sufficiently utilized [10, 11]. Furthermore, national guidelines can aid in the consistency and quality of health economics studies [12].

Over the years, different treatment strategies for AL have emerged, depending on the severity of the leakage and its clinical manifestation, but also on the surgeon’s preference, experience, and availability of interventional options [13]. Conventionally, AL is treated with stoma formation leading to faecal diversion with or without transanal or transgluteal drain placement, or acute salvage surgery with breakdown of the anastomosis and formation of an end colostomy [14]. More recently, active treatment strategies, such as endoscopic vacuum therapy (EVT) or the adapted endoscopic vacuum-assisted surgical closure technique (EVASC), have been increasingly employed because of their higher success rates in the re-establishment of a healed anastomosis [15, 16]. Although data on the success rates of these different treatment strategies are available, the differences in costs have not yet been explored. Cost analyses remain crucial to justify novel prevention and treatment strategies for AL.

This study aimed to provide a transparent cost analysis of different treatment strategies for AL detected within 30 days after restorative rectal cancer resection, specifically after total mesorectal excision (TME), within the first postoperative year.

## Methods

### Study design

This study was a cost analysis of patients included in the control cohort of the prospective IMARI-study. The IMARI-study is a multicentre clinical effectiveness trial, consisting of a control cohort in which routine practice for management of AL was registered, followed by an interventional cohort after implementation of a multi-interventional program for the prevention and treatment of AL [17]. The current analysis was based on patients included in the control cohort, in which the multi-interventional program had not yet been implemented as standard of care in the participating centres. Data within the control cohort were prospectively collected between February 2020 and January 2023 in 4 academic and 11 non-academic centres in the Netherlands. The IMARI-study protocol is registered with the Dutch Central Committee on Research Involving Human Subjects (registration number NL67600.018.18) and has been submitted to the http://www.onderzoekmetmensen.nl/en database (NL-OMON26456 and NL-OMON55903).

### Study population

Patients in the study were ≥ 18 years old and underwent a TME for one of the following indications: primary rectal cancer, regrowth of rectal cancer during a watch-and-wait approach or as a completion TME following local excision for rectal cancer. Patients were not included in the registry if no colorectal or coloanal anastomosis was created, if they had locally advanced rectal cancer requiring beyond-TME or multi-visceral resection or if they had undergone synchronous colonic resection. To achieve an even distribution of patients from centres across the Netherlands, the first 10 consecutive patients from each centre within the control cohort were selected. When fewer than 10 patients were available, all patients from that centre were included. Patients with late AL diagnosed more than 30 days postoperatively were excluded. Written informed consent was obtained from all study participants. The Medical Ethical Committee (METC 2019_055, 14 August 2019) of the Amsterdam UMC, location AMC approved the study.

### Definitions

AL was defined as the presence of any of the following factors, adapted from an earlier study [18]: contrast extravasation on imaging studies, or a peri-anastomotic collection requiring surgical or radiological or endoscopic intervention or a peri-anastomotic collection that either led to a delay in stoma reversal or led to resection or reconstruction of the anastomosis. Leaks were diagnosed using a computed tomography (CT) scan with rectal contrast or confirmed endoscopically in the operating room or endoscopy unit. All patients underwent a CT scan with rectal contrast 1 year after surgery. In the current analysis, only early detected ALs (diagnosed within 30 days after surgery) were included. The grading of AL according to the International Study Group of Rectal Cancer (ISREC) classification was added as Supplementary Table 1, whereas the cost analysis was stratified on the basis of the different treatment strategies employed.

Four treatment strategies for AL were defined in accordance with previous reports [19, 20]. Treatment strategies were categorized as active drainage (endoscopic vacuum therapy (EVT) with or without transanal surgical closure of the anastomotic defect, with a primary or secondary ileostomy), passive drainage (primary or secondary diverting ileostomy with or without transabdominal, transanal, or gluteal drain placement, abscess cavity washout or abdominal lavage), acute salvage surgery (immediate or delayed redo anastomosis or breakdown of the anastomosis with creation of an end colostomy) or no faecal diversion (without primary or secondary diverting ileostomy, treated with antibiotics only, transabdominal or gluteal drainage, surgical or endoscopic abscess drainage, abdominal or colonic lavage). Patients were initially assigned a treatment strategy on the basis of the initial treatment initiated after the diagnosis of AL. If patients switched to a different treatment strategy within 1 month, they were reassigned to the strategy they ultimately received for the cost analysis (e.g. from no faecal diversion to faecal diversion or salvage, or from faecal diversion to salvage).

### Outcome parameters

The primary outcome of this study was the incremental direct hospital costs for patients with early detected AL compared with those without AL over 1 year. The cost of the index procedure was not included in the cost calculation. The economic outcome measures used to quantify hospital costs were based on resource utilization and associated unit costs. Resource utilization was categorized into various components, including index hospitalization, (re)interventions, diagnostic imaging, endoscopy, ICU admissions, readmissions and outpatient visits. All data were prospectively recorded with a follow-up duration of 1 year after surgery.

### Economic evaluation

The analysis was conducted using the guideline for economic evaluations in healthcare of the Dutch Healthcare Institute [21]. The chosen perspective was the hospital perspective. The time horizon for this study was 1 year, starting from the index admission until 1-year postoperatively.

### Valuation of unit costs

The Dutch Healthcare Institute guideline compiles a public catalogue consisting of reference prices for healthcare activity. It is suggested to use the reference prices for each healthcare activity listed in this catalogue to enhance clear interpretation and comparability in economic evaluations. In cases where no costs were listed for units, such as for specific surgical interventions and other reinterventions, the unit costs were derived from a confidential file of the hospital costs ledger of the Amsterdam UMC. These unit costs are confidential and will therefore not be specified per unit. Unit costs in this study were determined by ‘bottom-up costing,’ which refers to estimating costs by summing individual components. This allowed for a detailed breakdown of the specific resources and services that were consumed. When applied to hospital costs, this method typically involves itemizing expenses for medical interventions, operations, hospitalization and diagnostics. The total costs are presented per patient in 2023 EURO (€). Cost data are often non-normally distributed and are skewed to the right. However, to accurately reflect actual expenses, costs are presented as the mean (Range = min.−max. difference). All available unit costs were corrected for inflation using consumer price index numbers available on Statline of the Central Bureau of Statistics (statline.cbs.nl) [22].

### Statistical analysis

Patient baseline characteristics were described using descriptive statistics. Tabulation shows numerical data as mean and standard deviation (SD) unless indicated otherwise. The costs were compared between the different treatment strategies. Chi-square or Fisher’s exact test was used to compare categorical data. The Mann–Whitney *U*-test was used for non-parametric data, and a *t*-test for parametric data. Statistical significance was determined using a *P*-value threshold of < 0.05. Statistical analyses were performed in IBM SPSS Statistics (v.28.0.1.1, IBM Corp., Armonk, New York, USA).

## Results

### Patient characteristics

A total of 32 patients with AL and 82 patients without AL were included in the cost analysis. The patient characteristics are summarized in Table 1*,* and are stratified according to the presence or absence of AL. In 44 of 114 (39%) patients, the TME procedure included the construction of a diverting stoma. Table 2 presents the diagnosis of AL in 32 patients, with a median time to detection of 5 days (interquartile range (IQR) 3.0–9.0). A diverting stoma was present in 13 (41%) of patients at the time of diagnosis, with variations observed across different treatment groups. Baseline characteristics were similar between the AL and non-AL patients, including the rate of diverting ileostomies, except for a significantly higher proportion of neoadjuvant therapy in the AL group (*P* = 0.012).

**Table 1:** Patient characteristics

	Total *N* = 114	Anastomotic leakage *N* = 32	No leakage *N* = 82	*P*-value^1,2^
Sex, male (%)	76 (66.7)	24 (75.0)	52 (63.4)	0.275*
Age at time of index surgery, mean ± SD (years)	60.2 ± 10.5	59.6 ± 10.0	60.4 ± 10.7	0.702**
BMI, mean ± SD (kg/m^2^)	25.3 ± 3.2	25.0 ± 3.1	25.3 ± 3.2	0.664**
Tumour distance from anal verge, median (IQR)	5.9 ± 6.7	4.7 (2.7–6.1)	5.2 (3.5–8.0)	0.172***
*ASA classification*				
ASA I	19 (16.7)	4 (12.5)	15 (18.3)	0.456*
ASA II	84 (73.7)	22 (68.8)	62 (75.6)	0.455*
ASA III	11 (9.6)	6 (18.8)	5 (6.1)	0.071*
ASA IV	0 (0)	0 (0)	0 (0)	-
Active smoker	13 (11.4)	4 (12.5)	9 (11.0)	0.755****
*Neoadjuvant therapy*				
None	57 (50.0)	11 (34.4)	46 (56.1)	0.037*
Short-course radiotherapy (5 × 5 Gy) only	25 (21.9)	12 (37.5)	13 (15.9)	0.012*
Short course radiotherapy (5 × 5 Gy) followed by chemotherapy	7 (6.1)	3 (9.4)	4 (4.9)	0.399****
Chemoradiotherapy	24 (21.1)	6 (18.8)	18 (22.0)	0.706*
Immunotherapy	1 (0.9)	0 (0)	1 (1.2)	1.000****
*Ostomy created during index surgery*				
Loop ileostomy	43 (37.7)	12 (37.5)	31 (37.8)	0.976*
Loop colostomy	1 (0.9)	1 (3.1)	0 (0)	0.281****
None	70 (61.4)	19 (59.4)	51 (62.2)	0.781*
*Comorbidities*				
No comorbidity	57 (50.0)	15 (46.9)	42 (51.2)	0.677*
Cardiac	16 (14.0)	4 (12.5)	12 (14.6)	0.768*
Peripheral vascular disease	16 (14.0)	3 (9.4)	13 (15.9)	0.371*
Pulmonary	18 (15.8)	8 (25.0)	10 (12.2)	0.092*
Diabetes	3 (2.6)	2 (6.3)	1 (1.2)	0.190****
Other	37 (32.5)	11 (34.4)	26 (31.7)	0.785*
*Surgical technique*				
Laparoscopy	72 (63.2)	18 (56.9)	54 (65.9)	0.339*
Robot	39 (34.2)	13 (40.6)	26 (31.7)	0.367*
Open	3 (2.6)	1 (3.1)	2 (2.4)	1.000****
+Transanal^3^	34 (29.9)	11 (35.5)	23 (28.4)	0.649*

*Chi-square test

** Independent-Samples *t* Test

*** Mann–Whitney *U* Test

****Fisher’s exact test

^1^Categorical variables are *n* (%) and *P*-value is derived from a Chi-square test or a Fisher’s exact test

^2^For numerical variables, the *P*-value is displayed after an independent *t*-test (for normally distributed variables) or a Mann–Whitney *U* test (for non-normally distributed variables)

^3^Transanal Total Mesorectal Excision (TaTME) in combination with laparoscopic or robotic abdominal phase

Abbreviations: *BMI* body mass index, *ASA* American Society of Anaesthesiologists, *TaTME* transanal total mesorectal excision

Bold values indicate a statistically significant difference

**Table 2:** Diagnosis of anastomotic leakage

	*n*	Median (IQR) days	Min–Max	Contrast study*, *n* (%)	Stoma at diagnosis, *n* (%)
All patients	32	5.0 (3.0–9.0)	2–20	25 (78%)	13 (41%)
Active drainage	18	6.5 (4.0–14.3)	3–19	13 (72%)	10 (56%)
Passive drainage	7	3.0 (3.0–9.0)	3–20	6 (86%)	1 (14%)
Salvage surgery	6	4.5 (2.8–7.3)	2–8	5 (83%)	2 (33%)
No diversion	1	5	5	1 (100%)	0

*Indicates patients diagnosed with anastomotic leakage via computed tomography (CT) with rectal contrast. Others were confirmed endoscopically in the operating room or endoscopy unit

### Resource utilization and costs of anastomotic leakage

The distribution of utilized resources and costs per patient indexed for 2023 is presented in Table 3. Patients with AL had a significantly prolonged mean index hospitalization of +9.6 days compared with non-AL patients (15.5 ± 13.5 versus 5.9 ± 6.5 days; *P* < 0.001). The number of ICU admission days was higher after AL, but not statistically significant (1.0 ± 3.5 versus 0.1 ± 0.3 days; *P* = 0.095). Utilization of diagnostics, readmissions, outpatient visits and reinterventions were all significantly higher in the AL group. Patients with AL underwent reinterventions more than eight times as frequently (6.0 ± 4.4 versus 0.7 ± 0.9; *P* < 0.001).

**Table 3:** Mean costs per patient per year, indexed for 2023

		Total *N* = 114		Anastomotic leakage *N* = 32		No anastomotic leakage *N* = 82			
	Unit	Mean unit (SD)	Costs in € (range)^1^	Mean unit (SD)	Costs in € (range)^1^	Mean unit (SD)	Costs in € (range)^1^	Mean cost difference (€)	*P*-value*
Hospitalization			8960.68 (56,814.00)		18,419.78 (52,306.00)		5269.31 (32,844.00)	13,150.47	< 0.001
Index admission	Days	8.6 ± 10.0	5524.84 (39,284.00)	15.5 ± 13.5	10,002.13 (39,284.00)	5.9 ± 6.5	3777.61 (22,540.00)	6224.52	< 0.001
Readmission	Days	4.0 ± 8.3	2598.60 (41,860.00)	9.0 ± 11.9	5775.88 (41,860.00)	2.1 ± 5.3	1358.68 (23,184.00)	4417.20	< 0.001
ICU	Days	0.3 ± 1.9	837.24 (49,086.00)	1.0 ± 3.5	2641.78 (49,086.00)	0.1 ± 0.3	133.02 (8181.00)	2508.76	0.095
Diagnostics			1165.11 (8312.72)		2389.42 (7868.46)		687.33 (4034.61)	**1702.09**	< 0.001
CT scans	Scans	1.9 ± 2.2	415.03 (2443.43)	3.9 ± 2.6	874.64 (2221.30)	1.1 ± 1.3	235.67 (1332.78)	638.97	< 0.001
Endoscopy	Procedures	1.4 ± 2.0	750.07 (6535.68)	2.8 ± 3.0	1514.78 (6535.68)	0.8 ± 1.1	451.65 (3812.48)	1063.13	< 0.001
Outpatient clinic	Visits	5.8 ± 4.6	722.23 (2864.88)	9.1 ± 5.3	1132.72 (2864.88)	4.5 ± 3.6	562.04 (1992.96)	570.68	< 0.001
Reinterventions^1^			4464.03 (19,031.46)		10,872.94 (19,031.46)		1963.00 (13,270.37)	8909.94	< 0.001
Surgical	Procedures	1.2 ± 1.6	3734.25 (19,031.46)	2.8 ± 1.9	8518.53 (19,031.46)	0.6 ± 0.8	1867.21 (13,270.37)	6651.32	< 0.001
Endoscopic	Procedures	0.9 ± 2.4	657.70 (1805.52)	2.8 ± 3.8	2159.42 (9315.36)	0.1 ± 0.4	71.67 (2064.33)	2087.75	< 0.001
Radiologic	Procedures	0.1 ± 0.5	72.08 (3290.76)	0.3 ± 0.9	194.99 (3290.76)	0.0 ± 0.1	24.12 (1977.70)	170.87	0.009
Total costs			15,312.04 (73,685.17)		32,814.86 (59,810.21)		8481.68 (41,600.26)	24,333.18	< 0.001

*Mann–Whitney *U* Test for numerical variables with a non-normal distribution

^1^Reinterventions both related and unrelated to anastomotic leakage

Abbreviations: ICU, Intensive Care Unit

The mean total costs per patient during the first postoperative year were €15.312 (min.−max. difference = €87.320). The mean incremental costs for the AL group were + €24.333 compared with the non-AL group (€32.815 (min. −max. difference = €59.810) versus €8.482 (min. −max. difference = €41.600), *P* < 0.001). This was equivalent to a 287% or a 3.9-fold increase in costs compared with non-AL patients. Hospitalization and (re)interventions were the main drivers of the incremental costs in the AL group, accounting for 54% (+€13.150) and 37% (+€8.910) of the incremental costs, respectively. The specifications of the employed (re)interventions for patients with AL are presented in Table 4.

**Table 4:** Interventions and reinterventions during 1 year

	Total *N* = 114	Patients with AL *N* = 32	Patients without AL *N* = 82
*Surgical interventions for AL*			
Surgical endosponge	14 (0.12)	14 (0.44)	–
Surgical drainage abscess	6 (0.05)	6 (0.19)	–
Transanal closure of anastomotic defect	14 (0.12)	14 (0.44)	–
Diverting ileostomy	10 (0.09)	10 (0.31)	–
Diverting ileostomy with endosponge	1 (0.01)	1 (0.03)	–
Diverting stoma with endosponge and colonic lavage	1 (0.01)	1 (0.03)	–
Breakdown of the anastomosis and permanent colostomy	6 (0.05)	6 (0.19)	–
Diverting stoma and immediate closure of the defect	1 (0.01)	1 (0.03)	–
Oversewing of the defect	2 (0.02)	2 (0.06)	–
Diagnostic laparoscopy	2 (0.02)	2 (0.06)	–
Redo of the anastomosis	1 (0.01)	1 (0.03)	–
*Endoscopic interventions for AL*			
Endoscopic endosponge	85 (0.75)	85 (2.66)	–
Dilatation anastomosis	1 (0.01)	1 (0.03)	–
Lavage abdominal drain	1 (0.01)	1 (0.03)	–
*Radiological interventions for AL*			
Radiological drainage abscess	8 (0.07)	8 (0.25)	–
*Interventions unrelated to AL*			
Ostomy reversal	51 (0.45)	21 (0.66)	30 (0.37)
Diagnostic laparoscopy	3 (0.03)	2 (0.06)	1 (0.01)
Surgical drainage abscess	3 (0.03)	2 (0.06)	1 (0.01)
Lower abdominal laparotomy	1 (0.01)	1 (0.03)	0
Abdominoperineal resection	1 (0.01)	1 (0.03)	0
Liver metastasis resection	3 (0.03)	2 (0.06)	1 (0.01)
Diverting ileostomy unrelated to AL	1 (0.01)	0	1 (0.01)
Dilatation anastomosis	1 (0.01)	0	1 (0.01)
Internal hernia repair	1 (0.01)	0	1 (0.01)
Sternotomy	1 (0.01)	0	1 (0.01)
Lichtenstein	1 (0.01)	0	1 (0.01)
Colonic lavage and drainage	1 (0.01)	0	1 (0.01)
Ostomy correction	1 (0.01)	0	1 (0.01)
Cholecystectomy	2 (0.02)	0	2 (0.02)
Ablation liver metastases	1 (0.01)	0	1 (0.01)
Proctoscopy	1 (0.01)	0	1 (0.01)
Radius fracture surgery	1 (0.01)	0	1 (0.01)
Stenosis resection of segment	2 (0.02)	0	2 (0.02)
Total pelvic exenteration	1 (0.01)	0	1 (0.01)
Neurectomy	1 (0.01)	0	1 (0.01)
Ureterolysis	1 (0.01)	0	1 (0.01)

Values are *n* (mean per patient). Abbreviations: *AL* anastomotic leakage. – not applicable

### Costs of different treatment strategies

**Fig. 1:**
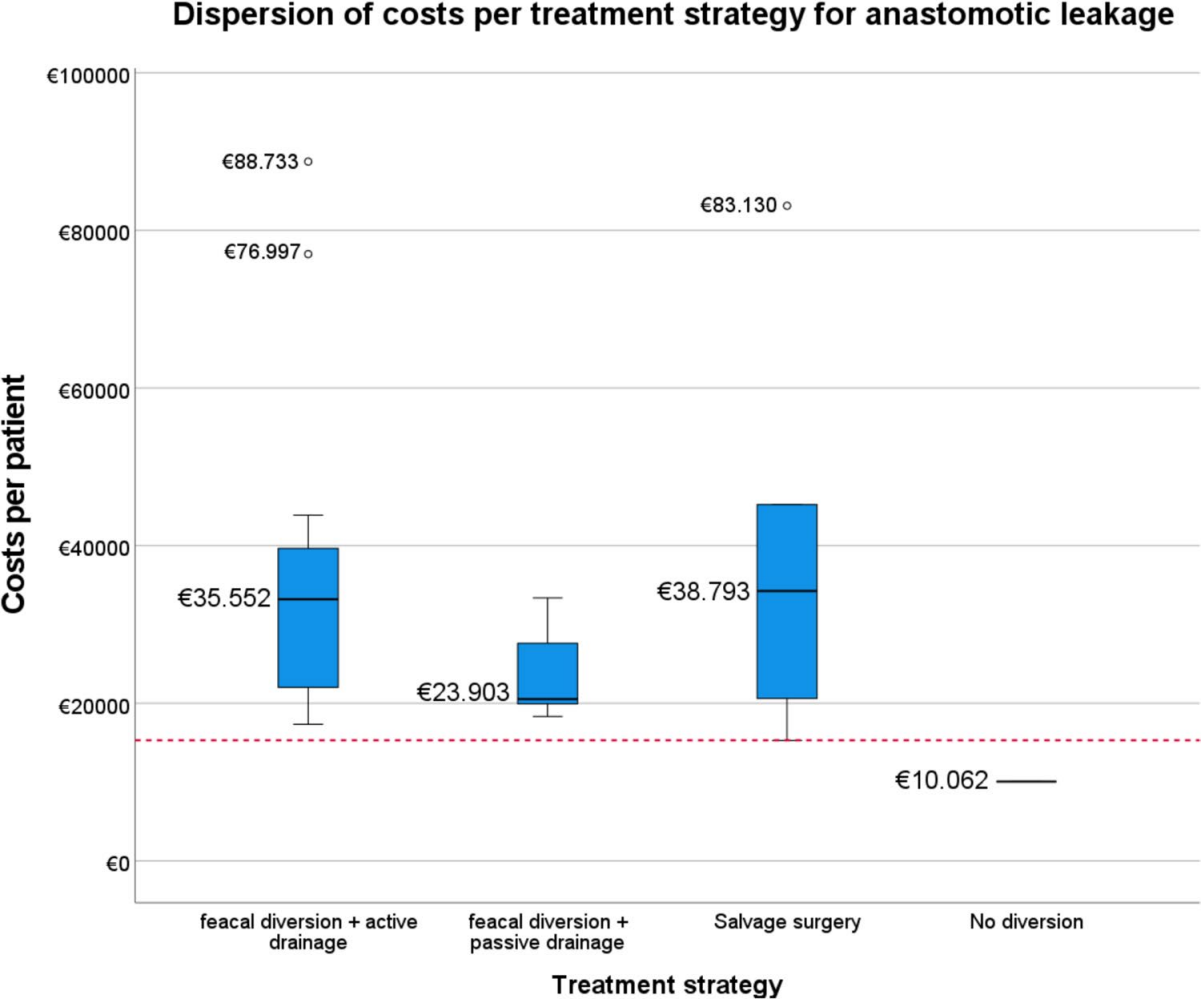
Dispersion of costs per treatment strategy for anastomotic leakage. Red dotted line represents the mean costs per patient in the entire cohort. NB. Three outliers were displayed separately because they fell more than 1.5 times beyond the first (Q1) or third (Q3) quartiles in the interquartile range (IQR)

The hospital costs stratified by different treatment strategies are presented in Table 5. Of the 32 patients with AL, 18 (56%) were treated with active drainage, 7 (22%) with passive drainage, 6 (19%) with salvage surgery, and 1 (3%) was treated without faecal diversion. The ISREC classification of the leaks by group is shown in Supplementary Table 1. Patients treated with salvage surgery accounted for the highest hospital costs of €38.793 (min.−max. difference = €67.842), whereas the patient undergoing no diversion incurred the lowest costs, totalling €10.062. The dispersion of costs for each treatment strategy is visualized in Fig. 1. The cost disparity was primarily driven by higher hospitalization and reintervention expenses. Salvage surgery incurred 321% higher hospitalization costs than no diversion (€24.388 versus €5.796; Δ = €18.592), 93% higher than passive drainage (€24.388 versus €12.604; Δ = €11.784), and 26% higher than active drainage (€24.388 versus €19.393; Δ = €4.995), primarily owing to prolonged index admission and ICU admission. Reintervention costs for active drainage were 26% higher than those for salvage surgery (€12.755 versus €10.145; Δ = €2.610) and substantially higher than those for passive drainage and no diversion (€12.755 versus. €8.210 and €0, respectively).

**Table 5:** Mean costs per patient per year, stratified by treatment strategy

		Active drainage *N* = 18		Passive drainage *N* = 7		Salvage surgery *N* = 6		No diversion *N* = 1	
	Unit	Mean unit (SD)	Costs in € (range)^1^	Mean unit (SD)	Costs in € (range)^1^	Mean unit (SD)	Costs in € (range)^1^	Units	Costs in €
Hospitalization			19,393.28 (63,663.00)		12,604.00 (11,592.00)		24,388.33 (65,830.00)		5796.00
Index admission	Days	15.7 ± 15.2	10,125.11 (39,284.00)	14.0 ± 7.2	9016.00 (12,236.00)	18.8 ± 15.4	12,128.67 (21,896.00)	3	1932.00
Readmission	Days	11.3 ± 15.2	7298.67 (41,860.00)	5.6 ± 3.6	3588.00 (6440.00)	6.3 ± 5.3	4078.67 (7728.00)	6	3864.00
ICU	Days	0.7 ± 2.2	1969.50 (21,816.00)	0	0	3.0 ± 7.3	8181.00 (7728.00)	0	0
Diagnostics			2461.92 (7868.00)		1790.46 (3267.84)		2848.51 (3633.09)		2522.44
CT scans	Scans	4.0 ± 2.5	888.52 (1999.17)	3.9 ± 2.6	856.79 (1332.78)	3.8 ± 3.5	851.50 (1999.17)	4	888.52
Endoscopy	Procedures	2.9 ± 3.5	1573.40 (6535.37)	1.7 ± 2.1	933.67 (3267.84)	3.7 ± 2.5	1997.01 (3267.84)	3	1633.92
Outpatient clinic	Visits	7.6 ± 4.1	941.12 (1992.96)	10.4 ± 7.5	1298.98 (2864.88)	11.3 ± 5.4	1411.68 (1992.96)	14	1743.84
Reinterventions^1^			12,755.46 (11,990.73)		8209.70 (6593.75)		10,144.67 (13,750.28)		0
Surgical	Procedures	3.4 ± 2.2	8752.66 (13,345.91)	2.3 ± 1.0	7739.59 (7831.75)	2.0 ± 1.1	10,144.69 (13,750.28)	0	0
Endoscopic	Procedures	5.1 ± 3.8	3838.96 (9135.16)	0	0	0	0	0	0
Radiologic	Procedures	0.4 ± 1.0	163.84 (2139.59)	0.4 ± 1.1	470.11 (3290.76)	0	0	0	0
Total costs			35,551.78 (71,396.56)		23,903.13 (15,027.73)		38,793.21 (67,842.37)		10,062.28

^1^Reinterventions both related and unrelated to anastomotic leakage. Abbreviations: *ICU* Intensive Care Unit

## Discussion

This multicentre study revealed that the incremental hospital costs for patients with AL averaged €24.333 per case in the first postoperative year. This accounted for a significant 3.9-fold increase in hospital costs compared with patients without AL. Patients with AL had significantly higher utilization of all resources except for ICU admissions. The primary drivers of the increased costs were hospitalization and (re)interventions. Different treatment strategies led to varying hospital costs, ranging from €10.062 for no faecal diversion, to €23.903 for faecal diversion with passive drainage, €35.552 for faecal diversion with active drainage and €38.793 for salvage surgery.

Several studies have evaluated the financial impact of AL after colorectal surgery in various healthcare settings, yielding divergent results. The common factor is that AL always significantly affects direct hospital costs, although the magnitude of this impact varies between studies, with mean incremental costs per patient ranging from €2.250 to €83.633 [7, 23, 24]. Heterogeneity in study design is a likely cause that can partially explain these differences. To illustrate this, Kumamaru et al. [25] calculated an incremental cost of €10.662 (converted to 2023 EUR) based solely on index admission costs, whereas Meyer [26] noted an incremental cost of €45.325 by comparing three AL patients with eight non-AL patients over a period of 5 years. Ensuring a sufficient time horizon in a health economic evaluation is critical for capturing all consequences of the condition of interest, in this case, the occurrence of AL [27]. Many costly (re)interventions occurred beyond the index admission or even well beyond the initial 90 days after surgery [28]. This indicates that a time horizon limited to the index admission is insufficient to capture the true financial burden. The costs after the first postoperative year can be attributed to the persistence of AL, which can progress to a chronic presacral sinus in a substantial proportion of cases and affect a large patient population. A previous study revealed that 48% of patients with AL experienced non-healing of the anastomosis after 1 year, thus evolving into chronic pelvic sepsis [18]. Our group recently estimated that pelvic sepsis treatment following AL incurs approximately €30.000 per patient, costs that may essentially also be attributed to AL [29].

This is the first study to distinguish hospital costs on the basis of four treatment strategies for AL. As inherent differences exist between the selected patients in each group, this study was not intended to demonstrate the superiority of one treatment strategy over another. Instead, the purpose was rather to highlight the financial implications of the various treatment pathways. The vast majority of AL cases in this cohort, consisting of patients who underwent TME, were confined pelvic leaks, for which the chosen treatment was driven more by the availability and experience of treatment options in specific participating centres rather than by the clinical presentation of the patient. This was depicted by the overall low rate of severe abdominal sepsis leading to ICU admission in this cohort. Furthermore, ICU admissions in this cohort did not differ between patients with and without AL. However, it should be emphasised that not all patients with AL are suitable for every presented treatment strategy. Patients with severe abdominal sepsis caused by intraperitoneal leakage due to ischemia of a colonic loop often require acute salvage surgery, whereas this is rarely required in patients with a confined pelvic leak. The higher hospitalization costs in the salvage surgery group were largely explained by prolonged ICU admission in a single case, indicating that a more severe manifestation of AL corresponded with the higher costs within this group.

Multiple reinterventions during the first postoperative year, along with the associated hospitalizations, were the primary cost drivers in the active drainage group. These costs were largely determined by the high frequency of endoscopic vacuum sponge changes, along with the related hospitalization expenses. Treatment strategies incorporating early initiation of active drainage have shown promising success rates, with a high rate of healed and functional anastomoses, even in severe cases of AL [16, 30, 31]. Thus, costs beyond the first year have the potential to decline compared with alternative, less effective treatment options, where there is a higher risk of developing chronic pelvic sepsis or requiring a permanent stoma. Direct and indirect costs of managing a colostomy over the first 5 years are estimated at approximately €126.600 (converted to 2023 EUR), and treatment costs for pelvic sepsis are estimated at nearly €30.000, also in 2023 EUR [29, 32]. Using these data in conjunction with the current findings, costs can be roughly extrapolated beyond the first postoperative year, with the total 5-year costs for these patients potentially reaching up to €180.000 if a leak leads to pelvic sepsis and a permanent colostomy. The sample size in the current analysis was insufficient to correlate the costs of specific treatment strategies with clinical success rates. Therefore, it remains unclear whether the relatively high treatment costs for active drainage in the first year are justified by the prevention of chronic pelvic sepsis and permanent stomas. The results from the IMARI-study [17], with prospective implementation of active treatment of AL, will provide further insight into this matter.

The findings of this study can be valuable for hospitals planning to implement active drainage for AL, and to gain insight into the financial implications of different treatment scenarios. It provides insight into short-term financial investments associated with various approaches, but care institutions must weigh this against the potential long-term benefits for patients, such as better physical, mental, and functional recovery, faster return to work and improved quality of life (QoL). Nevertheless, comparing different treatment options fairly in terms of cost remains challenging, as the presented strategies may not be applicable to each patient with AL. However, the severity of AL according to the current ISREC grading system does not always align with the treatment strategy chosen across centres. This is also why, in the current study, no cost stratification was performed on the basis of the ISREC classification, as the grading primarily reflects the chosen interventions used rather than the clinical severity, especially in confined pelvic leaks. For example, some centres without access to endoscopic vacuum therapy may be quicker to proceed with acute salvage surgery, which is inherently classified as a grade C leak. In centres with selective use of diverting ostomies during TME surgery and access to endoscopic vacuum therapy, leaks are more often classified as grade C, as creating a diverting ostomy is typically part of endoscopic vacuum therapy for AL, regardless of the patient’s clinical condition. The current grading of AL does not always correspond to the severity of the leak, but often reflects the hospital’s chosen therapy for AL. For example, in the current results, the passive drainage group mainly included grade C leaks, as six out of seven patients had no diverting stoma at the time of diagnosis but received an ostomy after AL detection. However, none of the patients in this group had severe sepsis leading to ICU admission. In addition, many of the mentioned indirect and societal factors that influence cost-effectiveness, such as productivity loss, non-hospital care and QoL, remain insufficiently explored. Further studies are needed to assess these factors and enable a fair comparison of treatment strategies.

A limitation of the current study is that we were unable to include all cost units in our calculations. The cost analysis was based on a prospective study in which the use of key resources relevant to the costs of AL were tracked. However, certain cost components could not be detailed comprehensively, such as specific laboratory tests beyond inflammatory markers and administered medications except antibiotics. This may have resulted in an underestimation of the true costs of AL. As hospitalization and reinterventions are known to be the main cost drivers, the underestimation is likely minimal. Another limitation of this study was the relatively small sample size, resulting in a limited number of patients per treatment strategy. In cost evaluation studies, there is a tradeoff between a smaller sample size with more detailed data on healthcare consumption and a larger sample size with less detailed data. This study opted for more detailed data, despite the lower sample size. Unlike other cost studies on AL that use larger national databases with limited data and tariff-based costing (e.g. reimbursement tariffs or Diagnostic-Related Group (DRG)/Diagnose Behandeling Combinatie (DBC)), this study employed individual unit costs derived from bottom-up costing methods [33–35]. These methods are considered to be more accurate in health economic evaluations than top-down costing approaches. Lastly, the cost analysis did not account for indirect costs resulting from extramural care or out-of-pocket expenses for patients and their families, nor did it consider costs related to QoL. These aspects remain underexplored and are an important focus for future studies to capture the full socioeconomic burden, extending beyond the hospital.

In conclusion, this study illustrated the magnitude of the financial burden caused by AL on Dutch hospitals after TME for rectal cancer. The incremental costs for AL averaged €24.333 per patient during the first year after surgery; however, the associated morbidity may extend well beyond this period. Among the different treatment strategies for AL, salvage surgery and active drainage incurred the highest costs. Future studies should evaluate how treatment costs relate to clinical success rates, including rates of chronic pelvic sepsis and permanent stomas, which will significantly affect costs after the first postoperative year.

## Supplementary information

Below is the link to the electronic supplementary material.

Supplementary file 1 (DOCX 23 KB)

## Data Availability

No datasets were generated or analysed during the current study.
