# Supplementary material for: Hospital costs of different treatment strategies for anastomotic leakage after total mesorectal excision: a multicentre cost analysis
Source: Tech Coloproctol. 2025 Oct 8;29(1):173. doi: 10.1007/s10151-025-03215-2 (PMC12507936; doi:10.1007/s10151-025-03215-2)
Supplement: Supplementary file 1 — (DOCX 23 KB) [file 10151_2025_3215_MOESM1_ESM.docx]

| Group | Grade A | Grade B | Grade C | Total cases |
| --- | --- | --- | --- | --- |
| Faecal diversion + active drainage | 0 | 10 (56%) | 8 (44%) | 18 |
| Faecal diversion + passive drainage | 0 | 1 (14%) | 6 (86%) | 7 |
| Salvage surgery | 0 | 0 | 6 (100%) | 6 |
| No diversion | 1 (100%) | 0 | 0 | 1 |

**Supplementary file 1. ISREC classification anastomotic leakage**

Values are displayed as n (% of total); ISREC, The International Study Group of Rectal Cancer
